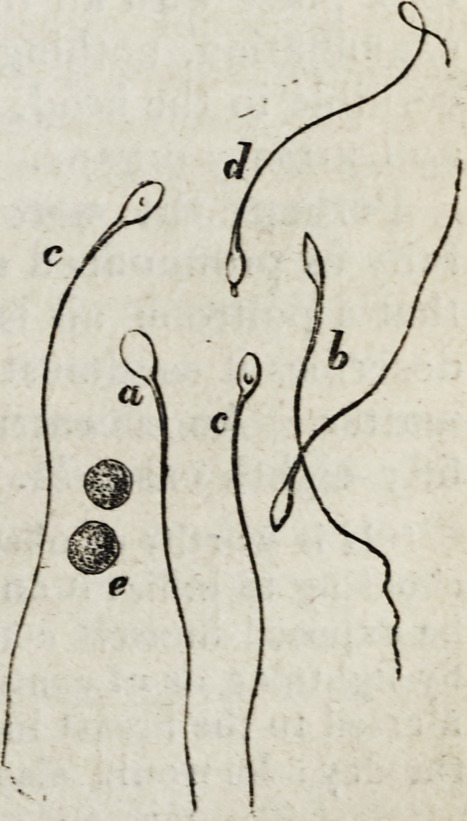# On Involuntary Seminal Discharges

**Published:** 1843-04

**Authors:** 


### Art. V.

1. *Des Pertes Seminales Involontaires.* Par M. Lallemand, Professeur à la Faculté de Médecine de Montpellier. *Paris,* 1836, 1838, 1839, 1841, 1842. Trois Tomes. 8vo, pp. 681, 550, 533.

*On Involuntary Seminal Discharges.* By M. Lallemand, Professor of the Faculty of Medicine of Montpellier.—*Paris,* 1836, 1838, 1839, 1841, 1842. Three Volumes.

2. *Mémoire sur l'Emploi des Caustiques dans quelques Maladies de l'Urètre.* Par le Dr. Civiale.—*Paris,* 1842. 8vo, pp. 59.

*Memoir on the Employment of Caustics in some diseases of the Urethra.* By Dr. Civiale.—*Paris,* 1842.

3. *De Vesiculis Seminalibus Dissertatio.* Auctore F. C. Faye, m.d. Medicos cohortis in exercitu Norvagico.—*Christianiæ,* 1841. 12mo, pp. 233.

*A Dissertation on the Seminal Vesicles.* By Dr. Faye, Regimental Surgeon in the Norwegian Army.—*Christiania,* 1841.

Most practitioners of any experience, more particularly those whose practice lies in large towns, not unfrequently meet with cases, for which the following may serve as a general picture: The patients are of the male sex, for the most part of early adult life; they are often pale in the complexion, slightly emaciated, sometimes haggard and sickly in appearance: their manners are shy and nervous, and they often have a remarkable air of timidity, we might almost say, servility and abjectness. They are at once restless and listless, and do not exhibit that cordial interest in persons and things, which naturally characterizes those not labouring under positive and palpable disease, or not chilled by age into apathy and obtuseness of feeling. If they come under the professional observation of the physician, they are usually found by him to be extremely reserved as to the origin of the derangements, physical and moral, of which they complain: their statements are frequently confused and even contradictory; and very often the causes which they allege as having primarily induced their complaints are at once discerned by the practitioner to be not at all adequate to account for the various and anomalous symptoms which present themselves. A marked form of complaint with them usually is—the impairment of the interest which they were used to feel in life; the disrelish of society, which has been stealing on them; the gradual loss of memory and of the capacity for clear and vigorous thought, which they have observed: perhaps also they talk of derangement of their digestive organs, of vertigo, of "lightness" of the head, &c; but they are in general (though not *always*) careful of approaching the real *fons et origo malorum;* although their studied avoidance of it may not at all have blinded the acute eye of him for whose advice they apply.

Although we shall not deny that many or even all the morbid phenomena now enumerated may not be owing to other causes than masturbation or involuntary seminal emission, as for example, to certain peculiar and rare affections of the nervous system, yet, in many or most cases, they will, on examination, be found to be due, directly or indirectly, to the former, namely, to sexual abuses or derangements. The patient may or may not admit the accuracy of the physician's suspicions: and while his acknowledgment of vicious habits removes, of course, all doubt from the practitioner's mind, his denial must not be too implicitly relied on; nor must induce the medical attendant to refrain from that line of treatment, nor from those warnings and suggestions, which his own observations and convictions incline him to adopt.

So far as the morbid phenomena which we have been describing depend simply on the practice of masturbation, it is plain that they may exist without any morbid condition of the genito-urinary passages; and that, accordingly, all that is necessary to the removal of these phenomena, is the abandonment, by the patient, of the vicious practice giving rise to them. No medical or surgical treatment is indicated or would be of any avail. But the case is different in regard to *involuntary* seminal emissions, whether the patient be conscious of the occurrence of these, or whether, as is sometimes the case, they happen in what may be called an *insensible* manner, and one that leaves him more or less ignorant of their existence; at least not cognizant of them, at the exact time that they take place. Emissions of this description appear to depend, almost invariably, if not constantly, on morbid states, inflammatory or nervous, of the genito-urinary ducts or muscles. We propose in this article to inquire into the nature and extent of these morbid conditions, and into the best modes of preventing or curing them.

The division of the subject which we propose is an exceedingly simple one; but perfectly suited for all practical purposes. We shall first consider the affections of the genito-urinary passages themselves, which give rise to involuntary seminal discharges. We shall next consider the other causes—generally extrinsic, and for the most part of a mechanical character, or else operating sympathetically—by which such discharges are occasioned. We shall then offer some observations on the best modes of treatment, local and general; and may sum up, if our limits allow us, with remarks on various miscellaneous matters, which do not naturally come within the line of *practical* inquiry, which we have just chalked out for ourselves.

A state of chronic inflammation, or at least of irritation or erethism, of the mucous membrane of the urethra, particularly at the prostatic portion of that canal, appears to be by far the most general cause of involuntary seminal discharges. We may here observe that this chronically inflamed or excited state often extends to the mucous membrane of the neck of the bladder, and down into the ejaculatory ducts and seminal vesicles. The causes of this condition of the portions of the genito-urinary mucous membrane now described, may be very various. The most frequent is the irritation caused by either inordinate coition or by masturbation; by gonorrhœa or by simple blenorrhagy, by phimosis, &c. But however different in *themselves*, the causes now enumerated and others are, they all, as giving rise to involuntary emissions, concur in one mode of operation; that, namely, of producing the local morbid state referred to, of the mucous membrane of the urethra, more particularly at its prostatic portion.

Now, the effect of all chronic inflammation of mucous membranes is the production of a mixed state of irritability and debility. Thus we see nausea and frequently vomiting result from a chronically inflamed condition of the gastric mucous membrane: diarrhœa and the premature transmission of undigested aliment take place in a similar condition of the intestinal mucous membrane; in like manner as a frequent and fretful expulsion of the urine is the sequela of an inflamed state of the inner coat of the bladder. In these cases, according to well-known laws of nervous sympathy and relation, the nerves of motion are excited to action in consequence of impressions made on the nerves of sensation. And in these and in all similar cases of morbid change, it is remarkable that a *less* than the normal stimulus suffices to call the affected part or organ into its peculiar action. Thus the bladder empties itself, while yet not distended to that degree, which, when the organ is healthy, causes a desire in us to evacuate it. The stomach rejects aliment, which, in other circumstances, would be mild, agreeable, and rapidly digested. And the severest tenesmus occurs, while the bowel contains nothing to be expelled.

Precisely in the same pathological manner as the one now described does a condition of chronic irritation of the mucous membrane of the prostatic portion of the urethra, and of the ejaculatory ducts and of the seminal vesicles, give rise to involuntary discharges of semen. The parts concerned in ejaculation are thrown into action with preternatural facility. A less quantity of semen than in a healthy state suffices to excite energetic venereal desires, and to prompt to their normal gratification, provokes, in the debilitated and irritable state of the parts, contractions of the seminal vesicles and expulsive efforts of the perineal muscles. Nor do these emissions of the semen extend merely to occasional nocturnal pollutions, which, in the absence of coition and in a healthy subject, are often not to be considered morbid; but they either degenerate into extremely frequent nocturnal discharges or even become diurnal; that is, take place when the patient strains at stool or empties his bladder; or when he takes the exercise of riding, or, in the worst cases, when he simply indulges in lascivious ideas.

We have said that involuntary seminal discharges depend *almost* constantly on a state of chronic irritation of the genito-urinary passages; and we believe that we are so far borne out in saying so by the fact, that although in certain cases involuntary emissions take place, without any disease of the urethra and spermatic ducts, this is comparatively rare. Yet it does occasionally happen. Thus strangled persons sometimes emit semen involuntarily; so do epileptics. In certain affections of the cerebellum and of the spinal cord, a like result follows. But our observation is, we believe, indisputable, that, in far the majority of cases, and perhaps even in those just enumerated, local irritation previously existing in the genito-urinary passages is necessary to give force to other less direct affections, such as piles, fissure of the rectum, dartrous eruptions of the perineum, so as to lead to involuntary seminal discharges. We have not met with any case, where the spermatic passages were in a perfectly sound state, in which the indirect causes just mentioned sufficed to produce involuntary emissions.

It is not necessary to occupy the reader's time in pointing out in what manner excessive coition, and still less masturbation, give rise to chronic disease of the spermatic passages. The mechanical irritation from without, the frequent and violent contraction of the ejaculatory ducts and vesiculæ, sufficiently account for the effect. All organs discharging their contents on mucous membrane, and having that membrane ramifying in their cavities, are subject, if frequently stimulated, to suffer hyperemic tumefaction, both in their substance and in the mucous membrane internally lining them. In other words, an organ too frequently called on to fulfil its peculiar function is apt to become hyper-vascular, and undergo either acute or chronic inflammation. To this cause, as well as to mechanical irritation and too frequently exerted contraction, must be ascribed the generally turgescent and inflamed states of the spermatic passages, brought on by inordinate coition and by masturbation, and giving rise to involuntary emissions.

The manner in which blenorrhagic or gonorrhœal inflammation leads to involuntary discharges is sufficiently obvious. The very cause on which these discharges depend, namely, irritation of the urethra and of the ejaculatory ducts, is here present and in emphatic operation. And we have only to suppose the chronic continuance of the gonorrhœal or blenorrhagic inflammation, and the consequent induction of morbid sensibility in the mucous membrane of the urethra, ejaculatory ducts, and vesiculæ seminales, to have all the conditions on which involuntary emissions depend.

Phimosis, as a cause of these emissions, acts by producing chronic inflammation of the glans and internal surface of the prepuce, which is propagated up the urethral canal. In phimosis there is usually a large secretion of sebaceous matter between the glans and prepuce, which either from the neglect of cleanliness or the actual impossibility of thoroughly removing it, acquires acrid properties, and causes inflammation and sometimes excoriation of the glans and prepuce. These, long continuing, induce irritation and ultimately inflammation of the urethra, with occasionally involuntary seminal discharges.

Severe chronic eruptions on the prepuce, scrotum, and glans, sometimes give rise to involuntary discharges of semen. This more especially happens when, as occasionally takes place, the affection which commenced on the skin actually extends itself up into the urethra. (Lallemand, tom. i. p. 226.)

Before noticing the affections of the rectum, which occasionally bring on involuntary emissions, we may remark, first, that disease of the prostate gland itself, and, secondly, chronic affections of the mucous coat of the bladder, and vesical calculus, may give rise to involuntary seminal losses. Cases have occurred in which, as a sequela of a blenorrhagia or gonorrhœa, the prostate has become the seat of chronic disease; in the course of which the gland has suppurated; the mucous membrane through which its ducts open into the urethra has become ulcerated, and the small sphincters of the mouths of the ejaculatory ducts have been eroded and destroyed. Hence a species of seminal incontinence and a tendency in the vesiculæ seminales constantly to allow their contents to escape. It not unfrequently happens that the inflammatory action, beginning in the prostate and involving, as has been now stated, the mouths of the ejaculatory ducts, extends into the vesiculæ seminales themselves, giving rise to various serious lesions. Sometimes the orifices of the vesiculæ become tumefied, so as to preclude them for acting as receptacles or diverticula for redundant semen; which, accordingly, accumulating in the vasa deferentia and ejaculatory ducts, is ever apt to be expelled on the slightest action of the ejaculatory muscles. In other cases pus forms, and, not escaping, concretes in the vesicles, and equally, with the occlusion of their mouths just referred to, unfits them from serving as diverticula for the semen. Again, as a result of the inflammatory action, acute or chronic, of which they may be the seat, the vesiculæ seminales occasionally become collapsed or rather contracted to a great degree.

As regards those morbid states which *begin* in the bladder and thence are propagated along the mucous membrane to the prostatic portion of the urethra and into the spermatic passages, leading ultimately to involuntary escapes of semen, M. Lallemand has remarked one or two circumstances of interest. He observes that morbid irritability of the bladder is usually accompanied by a similar state of the spermatic passages, and that the morbid predispositions which characterize the one, usually characterize the other. Thus those children who in infancy are troubled with irritability of bladder, who are compelled frequently to make urine and who are apt to do so when asleep, are usually, some ten or fifteen years later, the subject of a precisely analogous irritability or susceptibility of the spermatic organs, leading to either very frequent nocturnal pollutions or else to involuntary diurnal discharges. If they suffer from blenorrhagia, (and to this affection the persons in question are prone,) or from gonorrhœa, involuntary emissions are apt to establish themselves with a more than usual readiness. They are also more difficult to remove in such individuals, and are also more liable to recur.

Calculus in the bladder operates as a cause of seminal emissions, both by giving rise to chronic inflammation in the bladder and urethra, and also by exciting irregular and spasmodic actions in the perineal and ejaculatory muscles.

The affections of the anus and rectum which occasionally lead to involuntary discharges of semen, are fissure of the anus, hemorrhoids, diarrhœa, and the presence of ascarides in the bowel. It is obvious that these causes may operate in one of two ways; either by producing such a degree of irritation or inflammation as, after involving the anterior wall of the rectum, extends to the vesiculæ seminales and the upper part of the urethra, or else by inducing involuntary spasms in the perineal muscles and the muscles of the penis.

Among other local affections giving rise *occasionally* to involuntary emissions, may be enumerated hypospadias, hernia, varicocele. The effect in the first of these affections must be greatly due to the congenital largeness of the urethra and also of the orifices of the ejaculatory ducts, which almost always accompanies hypospadias; in the last two cases, the involuntary emissions must usually result from reflex nervous action caused by the rupture or varicosity, by which the muscles of the perineum and penis are excited to sympathetic action.

Certain states of the cerebellum, not of organic change nor even of a morbid nature, but consisting seemingly of a sort of physiological erethism, appear occasionally to have *predisposed* to involuntary discharges, and in other cases to the greatest and most immoderate *ardor copulationis.*

Lastly, a peculiar, perhaps we might say an idiosyncratic, sensibility of the nervous system in general, of that portion of the system distributed to the genito-urinary mucous membranes and organs in particular, may be the sole cause, at least the sole appreciable cause, of involuntary losses of semen, whether by nocturnal or by diurnal emissions.

We shall now a little more particularly describe the symptoms caused by involuntary seminal emissions, and in doing so we shall chiefly attend to *diurnal* discharges. Besides that nocturnal emissions cannot, in many cases, be regarded as morbid, their effects are not so disastrous as diurnal ones; while their diagnosis is easy, which is sometimes not the case with the other sort.

The occasions on which diurnal emissions most frequently take place are when the patient is at stool, and when he discharges his urine. In the former case the emission usually takes place at the moment that the patient strains violently. He may or he may not feel the passage of the semen; he may or he may not feel voluptuous sensations, at the instant of the accident occurring. Generally the semen escapes at the time that it is expelled from the seminal vesicles, and then the glans is found to be bedewed with a glutinous fluid, while, if the alvine evacuation is within reach of observation, a quantity of starch-like matter may often be noticed on or mingled with that evacuation. At other times or in other cases the semen, though expelled by the *vesiculæ seminales*, is not immediately emitted, but seems to remain for sometime in the urethra, from which it escapes when the patient is perhaps readjusting his clothes.

When the involuntary emission occurs during, and is caused by, the act of evacuating the bladder, it is usually when the last drops of the urine are expelled, and when the perineal muscles and the ejaculatory muscles of the penis, are called into particular exercise, that the seminal discharge takes place. In this, as in the other case, there may or there may not be slight voluptuous sensations. Usually the patient is sensible of the urethra undergoing a greater distention at the moment the semen passes than it had done previously during the passage of the urine; the sensation is as if something more bulky and more solid than the urine was passing along the urethra. If his eye chances to be directed to the stream of urine, he observes it suddenly and towards the end of the discharge, assume an opaque white or albuminous aspect, which, of course, is caused by the semen. This turbid state of the urine at the conclusion of the discharge cannot be confounded with opacity of the urine caused by vesical or urethral disease; because in the latter cases the turbid appearance of the urine occurs at the beginning and not at the end of that discharge. When the neck of the bladder or the urethra is the seat of any morbid discharge that discharge is by a physical necessity washed down or out by the *first* gush of urine. Or if the bladder generally is diseased then the whole stream of urine is turbid; but that the earlier part of the urine should be clear and the last drops accompanied with an albumen-like matter, is diagnostic of diurnal emissions.

The appearance which the semen presents in the chamber-pot is next to be described; and this is two-fold, namely, that of a starch-like liquor, occupying or constituting the lowest stratum of the contents of the vessel; or that of brilliant granules, like grains of the substance called semolina. We presume that these last can be nothing else than those portions of semen which have become inspissitated in the little cellular recesses of the seminal vesicles; and we are confirmed in this opinion by the fact, that when the emissions become very frequent, or after they have lasted long, the semen ceases to present the granular appearance, owing doubtless to its never being allowed to rest for any considerable time in the vesiculæ. It becomes aqueous in a greater and greater degree, and its presence in the urine is indicated by merely a slight cloudiness in that liquor. The granular appearances in the urine are distinguishable from those caused by salts by the fact of the former being visible in the urine as soon as it is voided, and not, as is the case with the latter, after it has cooled.

Before proceeding to notice with great brevity the functional and constitutional effects of involuntary seminal discharges, we shall first very shortly refer to the microscopic diagnosis of the semen; and secondly inquire whether M. Lallemand's opinions of the frequency of involuntary and more particularly of diurnal emissions be well founded.

Microscopic examinations of the semen may be useful in some cases, as, for example, in medico-legal investigations connected with a demanded divorce on the score of impotency; or in cases of royal or other important marriages, in which it is desired to ascertain whether progeny is or is not likely to be the result. When we wish to ascertain whether the semen be of a procreative quality, a single drop of the liquid which remains in the urethra after sexual connexion is sufficient. This being pressed out and placed on the object-glass, thousands of animalcules will, if the semen be normal, appear after a shorter or longer time. These in shape considerably resemble tadpoles, and move about with great vivacity. In pathologic cases, the zoosperms are sometimes of great transparency, so that they do not become visible until a certain stage in the process of evaporation. Sometimes the animalcules are in such numbers, that in order to observe satisfactorily the movements of particular ones, we must dilute the drop with water. If the globule of fluid be kept from evaporation, and at the temperature of the body, the movements of the animalcules may be observed in it for many hours.*

Animalcules are never found in the blenorrhagic or gonorrhœal discharge; globules of various sizes and shape, shreds of epithelium and filaments which appear very large under the microscope, which are cylindrical, more or less transparent, and sometimes branched, are alone discernible. These last consist of prostatic fluid, coagulated in the urethra; and to them, in affections of the prostate, is owing the turbid appearance of the *earlier* portion of the urine.

We are of opinion that M. Lallemand's notions as to the frequency of involuntary emissions are certainly exaggerated. He would have us believe that in all cases in which there are neither coition, manustupration, or nocturnal emissions, there *must* be diurnal discharges, of which the patient may or may not be conscious. Now, M. Lallemand seems to forget that the secretion of the testicles is a very different one from that of the liver or kidney, which last must *of necessity* be constantly going on and be constantly emitted. The excretion of the kidney has relation solely to the well-being of the individual; that of the testicles is correlative, and has regard to a condition external to and independent of the male; and as this is a condition not always present, it is but reasonable to suppose that nature provides for such a contingency by regulating the testicular secretion according to the actual wants or opportunities of the individual.

The functional and constitutional symptoms of involuntary discharges are next to be considered. The principal functional symptoms, or those which more immediately relate to the generative function itself, are more or less incapacity of erection, and consequently of impotence, or else a

* We may here remark, in general, that from page 385 to page 431 of M. Lallemand's second volume, there are given numerous directions and cautions as to the management of microscopy of the semen; which those interested in such inquiries and proposing to institute them, will do well to consult.

We here give a representation of the human zoospermata in their living healthy condition, taken from Wagner's Physiology, so excellently translated by Dr. Willis. "Spermatozoa (magnified from nine hundred to one thousand diameters) taken from the vas deferens of a man shortly after death; *a*, spermatozoon presenting the flat surface; *b*, ditto, viewed in profile, the margin being presented; *c*, ditto, presenting the circular spot on the surface, which some suppose to be a sucker; *d*, a spermatozoon exhibiting a process from the anterior extremity of its body, not unlike a proboscis; *e*, seminal granules." (p. 8.)

greater or less approach to it; feeble or too brief erection; emission either too hasty or too tardy, not to mention an aqueous, non-animalculised, non-procreative state of the semen, which renders impregnation impossible, even when coition is occasionally duly effected. To these may be added an unnatural absence of sexual desire.

Among the signs discoverable by surgical examination, are a great sensibility of the prostatic portion of the urethra, manifesting itself on the passage of a sound or bougie, which is often embraced spasmodically as it were by the urethra, and, if quitted for a moment by the hand of the operator, is jerked backwards and forwards in a singular manner.

As an occasional, though not a constant or an usually very striking symptom, is an irritability of the bladder corresponding to that of the vesiculæ seminales, and leading in the former to a morbid frequency of emptying the bladder, as in the latter to preternatural seminal discharges.

The constitutional symptoms of involuntary seminal discharges we have already, though very generally, adverted to, at the outset of this article. These symptoms are such as we might expect from a cause debilitating the nervous system generally, and not rarely mimic those of hypochondria. They consist of very anomalous derangements of the digestive organs of the most puzzling character; flitting and inexplicable pains in all parts of the body, (often, according to our observation, at the epigastrium, right hypochonder, and in the cardiac region,) numbnesses at various points, &c. We have also remarked on several occasions singular examples of loss of power in fingers, or in one of the upper or of the lower extremities, constituting a species of partial and temporary paralysis. Most of the patients of this description have complained to us of headach, which they usually describe as of a very peculiar kind; sometimes constrictive, sometimes expansive, or conveying the sensation of the brain being too large for the skull; sometimes as if there were a "burning" in the interior of the cranium. Headach indeed appears to be one of the most common of the *constitutional* symptoms; since we find M. Lallemand saying that it was through patients who came to consult him for supposed organic head affections that he was led to the study of involuntary pollutions, of which head affections, these pollutions were the real though unsuspected cause. And he gives one or two cases where death actually took place with all the symptoms of apoplectic effusion, but where, on examination, nothing adequately accounting for this catastrophe was discernible in the head, but the principal lesions were found in the spermatic and urinary organs.

Perhaps the *moral* symptoms of involuntary seminal emissions are fully as pronounced and as remarkable as the corporeal. We have said that a poltroon air is a very characteristic trait; indeed, M. Lallemand describes it as almost pathognomonic. This is, however, to overstate the matter. An eccentricity of manner and ideas is not uncommon. In his fifty-eighth case, M. Lallemand tells us:

"It is worthy of observation that the patient desired death, without, however, choosing to inflict it on himself, which is not rare in cases of this description; he exposed himself consequently to every storm, in the hope of being struck by lightning or of contracting some mortal disease; he traversed marshes, immersed to the breast in water, and retained his wet garments during the rest of the day; he would also have wished to have had a duel and to have died this way; but his companions regarded him as *malade imaginaire*, or as a fool." (Tom. i.)

Unsociality, suspicion, misanthropy, and we might also or rather say *misogyny*, or pointed aversion to the female sex, are almost invariably observed. A continued desire of locomotion, either in walking or in change of place and scene, is also a remarkable characteristic. From this last peculiarity, among other presumed proofs, M. Lallemand is disposed to regard Jean Jacques Rousseau as the permanent victim of involuntary seminal losses.* If so, the intellectual faculties in his case singularly escaped that impairment which usually accompanies the moral perversions just described; since, while those of the citizen of Geneva were always subtle and sometimes masterly in their manifestations, in most cases of this kind, they are confused, languid, feeble, indicating an incipient degree of hebetude or fatuity. The memory is usually remarkably impaired.

We now come to consider what is the best treatment of those involuntary seminal emissions, of the causes and symptoms of which we have been discoursing. We dismiss from our view nocturnal emissions, occurring with moderate frequency and when coition is abstained from, since such emissions, especially in young and robust men, cannot be considered morbid, nor are attended with hurtful consequences. The following observations on treatment are accordingly to be understood as applying to diurnal emissions, though they are likewise entirely pertinent to nocturnal discharges of very frequent recurrence.

The treatment is, like the symptoms, divisible into topical and constitutional, in discussing which we shall follow the same easy and natural method which we have endeavoured to do in the former part of this paper.

M. Lallemand's account of the circumstances which indicate the employment of cauterization of the prostatic portion of the urethra, (which as *one* of the modes of treating seminal emissions, we shall in due course consider,) may be quoted here, as giving a concise view both of the particular states to be treated and of the difficulties of treating these states:

"In cases of involuntary pollutions," he observes, "there exists almost always, at the same time, irritation and debility, excessive sensibility, and want of tone in the spermatic organs. .......... In almost all cases of diurnal pollutions, these two conditions may be observed to be present simultaneously, but in variable proportions; this precisely it is which disconcerts the practitioner and causes despair in the patient; since antiphlogistics and tonics, emollients and excitants, repose and fatigue, produce good or bad effects in the same individual, according as the irritation or the debility predominates at the moment." (Tom. i. p. 193.)

As cauterization, for involuntary seminal emissions depending simply on chronic irritation and inflammation, of the prostatic portion of the urethra and of the vesiculæ seminales is, or is alleged to be, the most efficient method of treatment, we may perhaps with propriety commence our observations with a consideration of its merits. And we shall begin with quoting M. Lallemand's explanation of its *modus operandi* in such cases:

* M. Lallemand seems to forget that Rousseau sent four or five children to L'Hospice des Enfans Trouvés.

"Cauterization," he observes, "has the advantage of combating at the same time these two orders of symptoms [debility and irritability]; by destroying the surface of the engorged tissues, it alters their morbid sensibility; the resolution of this state produces a contraction of the parts, which gives them new energy; hence it is that cauterization in the most of cases suffices to bring about a cure." (Tome i. p. 193.)

Without entering into an abstract discussion or inquiry in what peculiar manner it is, that nitrate of silver acts in restoring the normal sensibility and normal action to morbid surfaces, we may observe that the action of this agent in superseding or modifying what we may style the *natural* inflammation by its own artificially induced one; in taking down vascular tumefaction or morbid growth; in giving tone; in lessening irritability and sensibility, is now as well understood and as entirely established, as that of any surgical means whatever. Some of its beneficial action may doubtless be due simply to its destroying superincumbent morbid layers or parts, thereby giving nature an opportunity of replacing them by healthy ones; but much must also be attributed to its vital stimulation of the nerves and vessels. As we do not see that any useful end would be served, by discussing now and at greater length the *theory* of the action of nitrate of silver, we dismiss this part of the subject with the remarks just made.

As we believe it will be interesting and acceptable to our readers to know what has been the *actual* experience of *British* surgeons in this mode of treating involuntary seminal discharges, and as we have been put in possession, by private communications from some of the most eminent surgeons of this country, of the means of supplying our readers with such information, we shall proceed to transcribe extracts from some of the notes now lying before us; and having done this, and then given a short summary of the success of cauterization in M. Lallemand's own hands, together with an account of the manner of performing the operation, we shall afterwards consider *other* methods of treating involuntary emissions of semen. We must first, however, observe, possibly to the surprise of M. Lallemand, that in cases similar to, if not identical with, those in which he advises it, the armed bougie was applied to the urethra more than forty years ago by a British surgeon. This was pointed out to us by Mr. Curling. In one respect, the operation of Sir Everard Home differs from that of M. Lallemand, in so far as the former adopted cauterization for, and in employing it, believed himself to be treating, a "spasmodic stricture," although it is plain, from his own narrative of his cases, (the first of which we shall quote,) that no real stricture existed, but simply that irritable and morbidly sensible state of the prostatic portion of the urethra, which characterizes most or all of those cases for which M. Lallemand himself employs and recommends cauterization, and which renders the passage of the bougie at once painful and difficult.

"A gentleman who had early addicted himself to that pernicious vice (masturbation), had the following symptoms brought on at the age of twenty-one: frequent emissions in sleep, attended with great lassitude, depression of spirits, and loss of general strength, headach, inability to apply his mind to business, or exert himself for the whole day after such an event had taken place. These occasionally happened for several nights in succession, and then left him for six or seven, but that was the longest interval. The effects of these attacks upon his *reasoning faculties* was such as to make him completely miserable. I explained to him that I thought it probable that the symptoms of which he complained arose from a spasmodic stricture immediately behind the bulb of the urethra, brought on by the practices to which he confessed he had been addicted, and upon passing a bougie this proved to be really the case. As the urethra, although in an irritable state, admitted with some little difficulty a tolerably sized bougie to pass into the bladder, I thought the use of a common bougie might be sufficient to relax that part of the urethra, and by being frequently applied, might take off the preternatural sensibility. This, however, upon trial was found not to be the case, and little advantage was gained by its use. It was therefore found necessary to have recourse to the caustic, and six or seven applications of the armed bougie had the power to take off the sensibility of the urethra, as well as to remove the spasmodic contraction; and the tendency to emissions, with all their enervating effects, was very much relieved." (Practical Observations on the Treatment of Strictures of the Urethra, and of the Œsophagus. Edition 1803, Vol. ii. p. 247.)

The other case was one of *diurnal* emissions, in which "the moment his (the patient's) passions were excited, an effect very readily produced, emission took place, even before there was a complete erection," and there was also "a total inability to have connexion with women." Masturbation, in this as in the former case, was the inducing cause. The armed bougie was applied four times, but the amendment was only very partial. (Op. cit. p. 249.) One gentlemen writes us:

"I can recollect eleven cases in which I have found Lallemand's treatment successful, and one in which it did not completely succeed. In seven of the eleven cases a single application of the caustic was enough, in four it was necessary to apply it a second time; in a single case two applications were insufficient to cure the disease, although the improvement was very great. ........ The effects are immediate. A person in whom the discharge has continued for months, will have none for some days after the use of the caustic; but in some cases, as the irritation subsides, it will come again.

"I have carefully noted," writes another gentleman, "*twenty-seven* cases treated by the nitrate of silver, either applied in the solid form to the prostatic portion of the urethra, or used in the form of injection. Of these cases, thirteen were completely cured; eight so much benefited that the emissions only returned occasionally and produced but little effect on the system; and the remaining five benefited by the period between the emissions being lengthened, though not to the same extent as the cases in the second series. The application of the solid nitrate, in many cases, produces very great irritation, sometimes complete retention of urine for a short period; in others, inflammatory irritation with bloody urine, lasting from eight to ten days and even longer. These circumstances lead me to use solutions of the salt, one or two grains to the ounce, advising the patient to inject several times at intervals of three and four hours, till a marked irritation was brought on; then to discontinue the remedy for a time and to have recourse to it again if the cure was not effected, when the irritation has subsided. The injections used in this way produce often great irritation and bloody urine, which continues sometimes many days. They have, however, in almost every instance, a beneficial effect upon the emissions."

A well-known and experienced Scottish surgeon observes:

"With regard to Lallemand's method of cauterizing the urethra, I have tried it in above a dozen cases, and in the majority of them with decidedly good effects. In those distressing cases of irritability of the bladder, where the prostatic portion of the urethra is chiefly affected; in certain cases of chronic disease of the mucous membrane of the bladder, and in that very prevalent and debilitating complaint to which young men are subject, nocturnal emissions, the efficacy of the practice is sometimes very striking. In the latter case, when the cauterization of the prostatic portion of the urethra fails, I have been lately in the habit, from a knowledge of the very intimate sympathy subsisting between the parts, of applying the cautery to the external orifice of the canal, and for about an inch down, and I think, in some cases, with more decided advantage."

The last communication we shall quote is the following:

"I have employed the caustic in several cases in private and at the hospital, and I entertain a very favorable opinion of its efficacy: indeed, in some instances, its good effects have been quite remarkable. I have used it successfully in cases of seminal emissions from self-abuse, sexual excesses, or in cases of obstinate onanism, which affections appear to be attended with, and partly kept up by, a morbid state of the prostatic part of the urethra. After using the caustic, I have found benefit from small doses of cubeb powder (gr. x.—xxx.) combined with the tincture of hyoscyamus, and also from steel medicines; the patient practising, of course, the most rigid self-denial in respect to the cause of the complaint. The patients upon whom I have employed the caustic, have not experienced the severe effects described by Lallemand as 'occasionally' resulting from it. The application which I make is always slight and transient, and the effects of it subside in a day or two. I have never had occasion to apply leeches to the perineum afterward, and in no case has swelling of the testicles been produced by it. The hip-bath and rest is all the treatment usually required: but I am generally obliged to make two or three applications of the nitrate of silver, and sometimes more before the complaint is wholly removed."

In regard to caustic injections as practised by the gentlemen whose notes we have quoted second and third, we have ourselves had no experience in *this* complaint, though of course, in common with every practitioner, we have times without number ordered them in gonorrheal and blenorrhagic discharges. We are disposed to think that the former of these two gentlemen considerably overstates the effects of cauterization as they usually manifest themselves, and we are also disposed to believe that the application of the solid caustic to the deep part of the urethra is less irritating and more effectual than the application of it in solution and to the outer part of the urethra.

We had intended to quote some of M. Lallemand's cases: but as the details given in the communications of the gentleman which we have just transcribed, are complete, we content ourselves with stating that the reports of the Professor of Montpellier, making every allowance for the perhaps excusable partiality of their author, leave no doubt as to the useful effects of the operation of cauterization in properly selected cases and when prudently applied.

The space which M. Lallemand usually cauterizes, is from the neck of the bladder to the membranous part of the urethra; but sometimes he brushes over the internal surface of the bladder itself to a greater or less extent. He cautions us strongly against repeating the operation too soon; and advises us to wait two or three weeks before we re-apply the caustic. Many of his cases appear to have been cured by a single application. Pain, and a slight discharge of blood, but never amounting to hemorrhage, seems to have followed some of his operations; but these consequences disappeared at the end of from twelve to forty-eight hours. In one case they are described as lasting three weeks, but this is mentioned as a rare exception. When the emissions have been *diurnal*, M. Lallemand regards the conversion of them into *nocturnal*, and the fact of the emissions being once more accompanied with erections and with pleasurable sensations, as a sign of the favorable effects of treatment and prospect of cure.

Of the mode of performing that operation, and of the instrument for doing so, it is singular that all mention should have been omitted in the works of Lallemand which head this article. This somewhat unaccountable neglect, we must supply by the aid of Mr. Curling; whose paper, which we are about to quote from, well merits perusal.

"M. Lallemand's instrument, [which may be had of Mr. Weiss,] consists of a straight or curved platina canula or tube, rather smaller than a middle-sized catheter, through which plays a caustic holder; in the further extremity of which there is a narrow groove, eleven lines in length, for the purpose of holding the caustic. After filling the groove with the nitrate of silver, by fusing it over a spirit lamp, it becomes so securely fixed, that there is no longer any danger of its escaping. At the other end there is a sliding screw or stop, by which the action of the remedy may be limited to any extent less than the length of the groove which contains it. Another sliding stop affixed to the canula serves, after the distance of the meatus from the part to be cauterized has been ascertained, to prevent the instrument passing further into the canal." (Med. Gaz. Jan. 19, 1839.)

We may likewise quote the same gentleman's method of employing the instrument:

"I first pass," he says, "a wax bougie down to the stricture, in order to ascertain its distance from the meatus, which I mark with my finger-nail. I then regulate the caustic holder according to the extent of the urethra that I wish to touch, and having closed the instrument so as to conceal the caustic, I fix the sliding stop on the canula, at the exact distance from the extremity corresponding with the mark on the bougie. Having introduced the instrument thus prepared as far as the stricture, I push forward the caustic holder, and after rapidly making one complete turn, I instantly close the instrument and then withdraw it. It will be readily perceived that the great advantage of this instrument consists in its enabling the surgeon to limit the action of the caustic to the part diseased, and to as small an extent as he pleases. Thus, by rotating the caustic holder, the lunar stone may be applied to the whole circumference, to one side or to some particular part of the canal, while its action may be extended to any part within reach of the instrument." (Med. Gaz. Jan. 19, 1839.)

We shall now, with great brevity, advert to other modes than cauterization of treating involuntary seminal discharges, depending *solely* on chronic inflammation of the prostatic portion of the urethra and of the vesiculæ seminales. The daily introduction of a bougie, and the retaining it for a longer or shorter time in the urethra, may first be mentioned. This, as our own observation enables us to testify, is often useful. Leeching of and blisters to the perineum are means of no slight efficacy, especially where the prostate is tender and enlarged. We have also prescribed tartar-emetic frictions of the perineum with excellent effects. A total abandonment of masturbation and a moderate use, or even an entire though temporary disuse of coition, are, of course, indispensable measures. One of our correspondents states that he has found opiates extremely useful. They are so in most cases though not in all; since they sometimes seem to augment the disease. Conium is safer: and both it and opium may be used both constitutionally and as a suppository. Cold clysters are often of benefit. As regards general means, alcoholic and malt liquors must be abandoned. M. Lallemand's opinion of these is exceedingly hostile; and we believe he is right in this. The food should be nourishing, light, and unstimulant; the bowels should be of course attended to, and, as a general rule, country air and exercise prescribed. Among medicines and articles of diet, tea and coffee in excess, tobacco, camphor, nitrate of potass, aloes, must be abstained from.

We shall notice, with equal brevity, the modes of treating emissions dependent on other causes besides chronic irritation of the prostatic portion of the urethra. Phimosis requires circumcision; fissure of the rectum, section of the sphincter; dartrous affections, the use, internal and external, of sulphureous waters. If the bladder be the seat of chronic inflammation, which thence propagates itself into the urethra, thus causing emissions, that viscus may be cauterized. This is an operation not rarely performed by M. Lallemand, and with safety and success. If involuntary pollutions be caused or kept up by piles or ascarides, we need scarcely say that the appropriate means for the removal of these, must be had recourse to; nor is it necessary for us to indicate what these are. The same remark applies to hernia, varicocele, &c.

Lesions, organic or functional, of the spine and cerebellum require lines of treatment which it would, of course, be out of the question to enter on here: but we would make one general observation. Often, in cases in which the involuntary emissions have *originally* been solely or chiefly due to some cause extrinsic to sexual abuse, and other than chronic irritation of the prostatic portion of the urethra, these emissions do not cease immediately on the primary cause, whether phimosis, fissure of the rectum, ascarides, &c. being removed; but still continue, owing either to the ejaculatory muscles having acquired a *morbid habit* of contracting themselves, or else to chronic irritation having been set up in the urethra, and having survived the cause that originally produced it. In such cases, cauterization or some of the other means already indicated, must be had recourse to.

In many of M. Lallemand's cases, virility where there had been impotence followed marvellously soon after the operation of cauterization. But we must caution our readers against what we must view as somewhat unguarded representations of M. Lallemand on this head, and warn them that vigour of the generative organs, after a period of abuse, inaction, or disease, is not so readily nor so easily recovered.

On the subject of *Masturbation,* M. Lallemand gives a large amount of details, many of which are scarcely fitted to appear in an English dress, even although in the pages of a professional review.

In boys, this baneful habit is often excited by the practice of pulling forward the prepuce to relieve the irritation of stone or gravel. It is also sometimes involuntarily suggested to them by the habit of sleeping on their bellies, by which their genital parts are heated and pressed upon. Nurses should be made aware of this. It is astonishing at what an early age children may be initiated into this habit; M. Lallemand quotes a case of a child of eighteen months having been taught it by an abandoned nurse.

The prophylaxis which M. Lallemand proposes for self-abuse merits some attention. According to him, the first and surest means of curbing the tendency to it, whether in infants or adults, is *muscular exercise pushed to fatigue.* Our own observation leads us to the same conclusion: still, speaking philosophically, it must be admitted to be but a very imperfect and equivocal mode of meeting the difficulty. For, how can there be prophylaxis against the sexual appetite (this after all being the real question,) any effectual means other than the one pointed out and provided by nature, of satisfying a natural appetite? The answer is obvious.

We admit, indeed, that exercise is most important, muscular exercise being the natural antagonist of a too irritable and excitable nervous system; but, while we assent to M. Lallemand's proposal as the best, perhaps, of the two evils, while we willingly recommend its adoption in lieu of a better; we at the same time perceive that it is but a clumsy and inadequate substitute for the natural means. M. Lallemand himself, indeed, seems to be fully aware of this truth, since he concludes this part of his subject with the following remark:

"As to conciliating" [the gratification of] "these irresistible desires with the not less imperious requirements of society, I own that the difficulties are grave and manifold; but it is not by a system of inflexible rigour, that we shall arrive at a solution of the difficulty. It is necessary, that, sooner or later, laws and manners should accommodate themselves to the organization of man, by endeavouring to obtain from it the greatest possible advantage, to the interest at once of the individual and of the species." (Tom. ii. p. 264.)

In addition to muscular exercise as a preventive M. Lallemand suggests that the studies of young persons should be much more diversified, and should be rendered much more *engaging* than they at present are; that all the intellectual faculties, those that respect the fine arts, as well as those that respect science, should be actively and pleasingly occupied, in order that the attention and the thoughts of youth should be diverted from those more dangerous reflections and musings, incident to opening life, and which, in the author's opinion, the dry and harsh nature of scholastic study is so apt to favour.

We have left ourselves but little space to express the favorable opinion we entertain of Dr. Faye's small yet complete and perspicuous treatise. The following subjects are clearly but concisely and ably handled: the anatomy, physiology, pathological anatomy of the vesiculæ seminales; then the diseases or accidents to which these are liable, namely, inflammation, suppuration, induration, scirrhous degeneration, tuberculosis and scrophulosis, hemorrhagy, varicosity, calculus, fistula, atrophy, hypertrophy. Next, the etiology, symptomatology, progress, duration, and termination, the diagnosis, prognosis and treatment of the above affections, are well and judiciously, though succinctly discoursed of. The following, Dr. Faye conceives, are the uses of the vesiculæ, respecting which, our readers are aware, there have been and still are discordant opinions, among the most eminent physiologists.

1. "The vesiculæ receive the semen secreted by the testicles, which they partly retain, partly absorb, by a peculiar property of their internal membrane.

2. "They secrete some mucous fluid, which enters into intimate commixture with the semen received from the deferent vessels.

3. "They act a principal (?) part (maximas partes,) in ejaculating the semen.

4. "They have no insignificant share in stimulating and sustaining the sexual instinct." (pp. 25, 44.)

We may add that the anatomy of the seminal vesicles given by Dr. Faye is derived from a *personal* examination of thirty adult subjects.

The latin, in which Dr. Faye's treatise is written is tolerably pure, and is not inelegant.

We had here, as we thought, completed this article, when we received the third and last volume of M. Lallemand's treatise, which is only just published, and also, the pamphlet of M. Civiale which we believe is only printed for private distribution. About the same period an important communication on the subject of the article by Mr. Phillips made its appearance in the Medical Gazette.*

The appearance of these publications induces us somewhat to extend our remarks. The monograph of Civiale is the most important of the three, and that, accordingly, on which we shall bestow our chief attention. We shall, however, in the first place, make a few observations on the relatively very voluminous " tome" of M. Lallemand.

There seems to us to be in this more than in any of the preceding volumes of M. Lallemand on the same subject, more decided symptoms of mere book-making; and a more obvious tendency to exaggerate the importance of the subject treated of. There is little or nothing essentially new in this last volume; little or nothing which has not only been referred to, but even *sufficiently* discussed in one or other of the former volumes on seminal discharges. In short, the present publication may be described as a mere paraphrase of parts of the preceding ones; those portions of it which have any claims to novelty, comprising a very small part of the volume, and that the least important.

In the third volume, the " general symptoms" which characterize, or are induced by involuntary seminal discharges, are the ostensible subject of discussion. We shall simply enumerate a few of these ; infecundity, fever, disorders of digestion, nutrition ; affections of animal temperature, (caloricité,) calvities, affections of normal "phonation," lesions of respiration and circulation, of innervation, under which head are treated, " myotility," sensibility, gustation, olfaction, audition, vision, &c., with notices of the morbid changes produced in these, by the discharges in question. In all this part of the volume, and in the succeeding portion, on to the 238th page, in which various alleged "general affections," (many of which appear to us mere modifications of each other,) are described, there is nothing which does not appear to us to have been adequately discoursed of previously. The " treatment" which extends from page 241 to page 470, is hardly anything else but a repetition of the means, local and general, already amply adverted to by the author.

The subject of prophylaxis is next again treated of; and the following additional remarks introduced at page 487, we shall quote, as they precisely confirm the opinions we have ourselves expressed in a former paragraph :

"However the most efficacious of these coercive measures cannot perfectly succeed except with infants. After puberty, the secretion of semen cannot be suspended or even sensibly diminished by any means compatible with health ; the accumulation of this fluid in the seminal vesicles must accordingly produce importunate erections, inevitable relapses, or else nocturnal and diurnal pol-

* Observations on Seminal and other Discharges from the Urethra; with illustrative cases. By Benjamin Phillips, F.R.S., Surgeon to the St. Mary-le-bone Infirmary, &c. —London Medical Gazette, Nos. xiii and xvii, January, 1843.

lutions; all which cannot be prevented, save by sexual intercourse. This means chiefly it is that can recall to the normal state the deteriorated functions, and modify the perverted sensibility of the organs. It is the sole really effectual means both for the present and the future, the only one that can completely reform unnatural tastes, by making the patient perceive the immense distance which separates his melancholy pleasures from those physiologically procured."

The volume closes with some reflections of considerable interest on the political causes, consequences, and preventives of masturbation; and on the due regulation of the sexual appetite. Having already stated that we must decline, on the present occasion, entering upon the theoretical branch of the subject, and on the highly important questions in regard to social economy and public morality, connected with or resulting from the sexual instincts and relations, we pass without further remark to the pamphlet of M. Civiale.

The small treatise of this distinguished lithotritist is principally directed to the consideration of the effects of cauterization in cases of *stricture;* consequently, it does not properly come within the scope and design of the present article. Towards the end of the pamphlet, however, the author treats of cauterization as applied to the cure of urethral discharges; although he does not, so far as we can perceive, intend that *seminal* emissions should be comprised under that term. Hence no small part of the objections of M. Civiale do not apply altogether to M. Lallemand's praises of cauterization as used in spermatic discharges. We willingly admit that M. Lallemand over-rates the advantages of cauterization; and that, besides it, many means are requisite to the removal of involuntary seminal discharges, and to the restoration to the genital organs of their due actions and vigour; such as abstinence from masturbation; moderation in venery where it has been excessively practised; the adoption or more frequent use of it, where it has been too little engaged in, &c.; still we hold the opinion already expressed, that in the commonest cases of seminal discharges, depending on excited sensibility and chronic vascular irritation of the mucous membrane of the spermatic passages, cauterization is undoubtedly one of the most efficient curative means; nor do we find in M. Civiale's monograph anything to invalidate our persuasion.

Still the opinion of so experienced and enlightened a practitioner as M. Civiale allowedly is, must always carry weight with it; and we think it proper to quote the following passage from his pamphlet, in which the difference of his experience in cauterization and that of its partisans, is accounted for on grounds which, if well founded, would tend considerably to modify the otherwise favorable opinion which we might be inclined to entertain of it. We are bound to state, that in our practice we have not met with the inconveniences referred to by M. Civiale; although we have had an eye for considerable periods of time on the subjects of operation; and as we rather suppose, from the tenor of his remarks, that M. Civiale refers to cauterization of the urethra generally, not of that part where the prostate is situated, and where cauterization may be practised without the same risk of the ill consequences alluded to by M. Civiale, as if the operation where performed on the anterior parts of the canal, this further circumstance must be kept in mind in considering his objections.

After informing us (page 54,) that " the greater part of the patients whom he saw, had not been cauterized by him, but had sought his aid in order to be delivered from the sufferings which cauterization had caused them;" he goes on to say:

"Thus, the facts presented in my practice are not in favour of cauterization of the urethra and of the neck of the bladder. They contrast so strangely with those which other practitioners have collected, that I think it useful to indicate the cause of this difference. One might suppose that the facts are contradictory; that there is error or mis-statement in the returns of one party or the other; and yet there is not. A man contracts a blenorrhagia; the disease persists; various simple means are employed without benefit. Nitrate of silver is applied, and the discharge ceases. The patient is lost sight of, and is reported cured. He is so in fact, in so far that the discharge has ceased to exist...... but at the end of some months, slight difficulty in making water is perceived; there are irritation, smarting, sensibility in the testicles, often also a discharge. Demulcents are employed; the state of matters is relieved, and the patient is satisfied. In all the cases, when he feels the necessity of calling in the aid of art, he applies, either to his ordinary medical attendant, or to surgeons celebrated for the cure of the affection by which he is attacked; seldom does he return to the practitioner who treated the blennorrhagy, and that for different reasons easy to conceive. The partisan of cauterization, therefore, has but few opportunities of observing the secondary effects of the means which he employs. He acts *bona fide,* in announcing his success; but this *bona fide* does not prevent him from being deceived." (pp. 56-7.)

The author goes on to remark that the same observations apply to the use of caustic in cases of stricture; which affection, (be it here noticed,) Civiale seems to be of opinion, is always better treated by dilatation than by cauterization; nay, he even maintains that the latter method is and must be, in *every* case, either impracticable or useless: impracticable, if the stricture is so narrow as not to admit of the passage of dilating instruments; since in this case the caustic cannot be applied to the morbid excrescence or callous contraction itself (causing the stricture,) but to the part or parts *anterior* to these; useless, since if a *porte-caustique* can pass, so might a simply dilating tube, which being the case, it follows, according to Dr. Civiale, that the case might be treated without caustic.

In confirmation of the views above quoted, as to the occasionally disastrous results of cauterization in cases of urethral discharge, Dr. Civiale cites three cases which had presented themselves to him on the same day.

"One," he informs us, " had been under treatment, during eighteen months, by a skilful surgeon of Paris. He had been successively scarified and cauterized. This unfortunate person, of a very irritable temperament, dragged on a painful existence; his genital organs were become so sensible, as not to be able to endure the contact of the patient's clothes. Another individual came from London, where he had been subjected to antiphlogistics, to cubebs, to balsam of copaiva, and, finally, to numerous cauterizations; his genital organs were not excessively painful, but the walls of the urethra had acquired such hardness and roughness; the surface of the canal was at the same time so irritable, that the emission of urine was very painful; treatment of three months' duration was required to re-establish him. The third was a young physician, to whom the perusal of certain works on strictures of the urethra, had proved disastrous. For a simple uretritis of some days' date, he had been subjected to injections with a solution of nitrate of silver; and although there had not been above a quarter of a grain of the salt to the ounce of water, or, as M. Deberrey would say, for that very reason, there had manifested itself, in the deep part of the urethra, and at the neck of the bladder, pains dull but excessively obstinate; the discharge had disappeared, but the pains, which extended to the sacrum and pubis, had plunged the patient into such a state of melancholy, that I was not without fears for the result. At the end of some weeks, he quitted Paris, and I have seen him no more." (pp. 55-6.)

It must not be inferred from the above quotation, that Dr. Civiale means to represent the application of caustic as a generally severe or an always objectionable treatment. On the contrary he asserts that, according to his experience, the operation, as regards severity, does not merit the strong terms occasionally used by M. Lallemand himself. This result he indeed partly accounts for from the fact that in many cases both of stricture and of seminal emissions, in which caustic is applied or supposed to be applied to the urethra, no such application actually takes place; the caustic being dissolved in the urethral mucosities and never coming in contact with, or only very imperfectly acting on, the mucous membrane; and he seems to be of opinion that when the caustic does come in contact with the mucous membrane, the pain is *always* very acute. He discusses the question whether the caustic is best applied in the solid or liquid form; and alleges in behalf of the latter method that it has a real advantage over the mode of cauterization proposed by M. Lallemand, that, namely, of acting in "a manner more uniform and more general; so that, if by the former mode, healthy parts are attacked as well as morbid ones, at least these last are not passed over, a circumstance which may happen when one pencils the walls of the bladder." (p. 523.) We must also find room for the following final quotation:

"Let me be permitted to add one reflection. The partisans of cauterization of the neck of the bladder are not agreed amongst themselves as to the sensation which it produces. The pain is slight according to some; acute, according to others. It is even possible that it may persist, and that accidents may hence result. Hence attempts have been made to combat and prevent the consequence of the cause which provokes it (the pain). As it has been attributed to the loss of substance, to the ulceration determined by the caustic, attempts have been made to favour the cicatrization of the surface acted on, by applying a cerate in which is incorporated a small quantity of acetate of lead and of opium." (p. 48.)

After pointing out the "impossibility" of efficiently applying this cerate, M. Civiale adds:

"Moreover, into what strange contradiction do they fall who tell us that the nitrate of silver is of such marvellous efficacy in removing those ulcerations to which they ascribe obstinate urethral discharges, yet who, on the other hand, represent it as itself the source of ulcerations causing grave consequences, which they try to obviate by impracticable applications of cerate." (p. 49.)

Dr. Civiale's monograph is written with his usual facility and his usual talent; it contains in a short space much practical information and is plainly the production of an enlightened, skilful, and experienced practitioner; it adduces some facts and details and observations which must modify, in some degree, both the use and the reputation of cauterization; still the pamphlet, partaking somewhat of the character of a formal philippic against cauterization and M. Lallemand, is to be estimated accordingly, and Civiale's statements are to be received with the same reservations with which those of the professor of Montpellier ought to be. From the facts and arguments adduced in the course of this article, every judicious practitioner will be enabled to form a tolerably fair judgment of the merits of cauterization.

As we find that in the preceding pages, which were written before the appearance of Mr. Phillips's papers in the Medical Gazette, we have adverted to all the facts and observations which he has so seasonably given to the profession, we shall make no further remark in regard to the papers referred to, except to express our opinion of their value and to recommend them to the reader's attention. There is, indeed, one direction given by Mr. Phillips to which we may call attention. He says:

"When the primary cause of the affection has ceased, it is necessary to examine the urethra with an exploring instrument; and for the purpose I prefer an elastic catheter. The point where the pain is most acute must be accurately noted. The instrument must then be passed on carefully until urine passes along it. Observe how far it has penetrated, and having noted this, you must arrange your caustic apparatus so that it shall not reach so far by an inch, because the prostatic portion of the canal is not commonly implicated in the irritation. The point upon which the caustic is to be applied is, as near as practicable, about the region of the orifices of the ejaculatory ducts." (Med. Gaz., Jan. 1843.)

We must own that we attach little practical importance to this caution, and are of opinion that Mr. Phillips will find, that according to his own plan of cauterization, nearly the same point of the urethra is operated upon as is done when the method described by Mr. Curling and already quoted, is had recourse to.

In conclusion, we would make two observations of a somewhat desultory nature. The first is, that, in perusing the last volume of M. Lallemand, we have been astonished and displeased, we had almost said disgusted, at the inconsiderate endeavour of the author to father almost every symptom, every ailment which individuals may labour under, on involuntary seminal emissions, the consequences of inordinate venery or still more vicious habits. If M. Lallemand were to be credited, every man who has a melancholy or abstracted air, who is hypochondriac, who is somewhat unsocial, who, with unusual care, eschews vinous or spirituous drinks, &c., *ought* to be suspected to labour under involuntary seminal discharges! What an imprudent and injurious and ridiculous exaggeration is this! We know persons who not only exhibit all the symptoms we have just enumerated, but who have (what M. Lallemand believes to be impossible) a nearly total disrelish for sexual intercourse, yet without having either nocturnal or diurnal emissions, and who, moreover, never practised self-pollution. We have at this moment patients such as we describe under our care; and we therefore feel indignant at the attempt of M. Lallemand to refer every innocent appearance of mental or of physical languor to a malady, the ordinary and most frequent causes of which are of an opprobrious and disgraceful kind. The tragical fate of Delpech, the late distinguished surgeon of the same city in which Lallemand himself practises, ought to be a warning to him not rashly to indulge in such surmises.

Our second remark is, that perhaps the tendency to frequent expulsions of the semen which follows continued masturbation or excessive venery long practised, may be accounted for not solely from a state of chronic irritation and morbid sensibility in the seminal ducts and vesicles, but also from an actual diminution of the size of these, and, consequently, of their power of containing beyond a certain and that a less than the normal quantity of spermatic fluid. We know that persons, boys for example, in the habit of frequently emptying their bladders, at length cannot contain more than a small quantity of urine; the bladder having most probably become somewhat contracted, simply because it has been seldom—for a greater or less length of time—called upon to contain an ordinary quantity of urine. If this view of the source, in part, of seminal discharges be well founded, (as we rather suspect it to be,) it has practical consequences as regards treatment. It explains the slowness and gradual nature of the cure; the tardiness with which the spermatic receptacles regain the power of containing a normal amount of semen; it encourages perseverance both in the patient and practitioners. Possibly, the beneficial effect of nitrate of silver applied to the orifices of the seminal ducts is in part due to its astringent action on these, and its thereby *compelling*, as it were, the diverticula to admit a larger supply of semen, and to retain it for a longer time, than they were lately used to do.

---

## Figures and Tables

**Figure f1:**